# Investigating Cox-2 and EGFR as Biomarkers in Canine Oral Squamous Cell Carcinoma: Implications for Diagnosis and Therapy

**DOI:** 10.3390/cimb46010031

**Published:** 2024-01-04

**Authors:** Rita Files, Catarina Santos, Felisbina L. Queiroga, Filipe Silva, Leonor Delgado, Isabel Pires, Justina Prada

**Affiliations:** 1 Department of Veterinary Sciences, University of Trás-os-Montes and Alto Douro, 5000-801 Vila Real, Portugal; ritafiles2000@gmail.com (R.F.); catarinasls13@gmail.com (C.S.); fqueirog@utad.pt (F.L.Q.); fsilva@utad.pt (F.S.); jprada@utad.pt (J.P.); 2 Animal and Veterinary Research Centre (CECAV), Associate Laboratory for Animal and Veterinary Sciences (AL4AnimalS), University of Trás-os-Montes and Alto Douro, 5000-801 Vila Real, Portugal; 3 Centre for the Study of Animal Science, CECA-ICETA, University of Porto, 4200-427 Porto, Portugal; 4 UNIPRO—Oral Pathology and Rehabilitation Research Unit, University Institute of Health Sciences—CESPU (IUCS-CESPU), 4585-116 Gandra, Portugal; mleonor.delgado@iucs.cespu.pt; 5 Pathology Department, INNO Serviços Especializados em Veterinária, 4710-503 Braga, Portugal

**Keywords:** oral squamous cell carcinoma, EGFR, COX-2, histological grade, canine

## Abstract

Oral squamous cell carcinoma (OSCC) is a common and highly aggressive dog tumor known for its local invasiveness and metastatic potential. Understanding the molecular mechanisms driving the development and progression of OSCC is crucial for improving diagnostic and therapeutic strategies. Additionally, spontaneous oral squamous cell carcinomas in dogs are an excellent model for studying human counterparts. In this study, we aimed to investigate the significance of two key molecular components, Cox-2 and EGFR, in canine OSCC. We examined 34 tumor sections from various dog breeds to assess the immunoexpression of Cox-2 and EGFR. Our findings revealed that Cox-2 was highly expressed in 70.6% of cases, while EGFR overexpression was observed in 44.1%. Cox-2 overexpression showed association with histological grade of malignancy (HGM) (*p* = 0.006) and EGFR with vascular invasion (*p* = 0.006). COX-2 and EGFR concurrent expression was associated with HGM (*p* = 0.002), as well as with the presence of vascular invasion (*p* = 0.002). These data suggest that Cox-2 and EGFR could be promising biomarkers and potential therapeutic targets, opening avenues for developing novel treatment strategies for dogs affected by OSCC. Further studies are warranted to delve deeper into these findings and translate them into clinical practice.

## 1. Introduction

With the rapid developments in the field of veterinary oncology, there is a great need for a better understanding of the molecular alterations behind the development of animal cancer [1]. Thus, the oral cavity is one of the most frequent sites of canine neoplastic proliferation, accounting for around 5 to 7% of tumors in dogs [1].

In dogs, oral squamous cell carcinoma (OSCC) is the second most prevalent malignant oral epithelial neoplasm (17% to 25%) [1]. Oral squamous cell carcinomas in dogs predominantly appear on the gingiva, affecting both the upper and lower areas, the tongue, and the tonsils. These carcinomas are also found in the lips, hard or soft palate, and the pharynx. Biological behavior depends on their location. Generally, oral tumors in dogs have a 15% rate of metastasizing, while those on the tongue can exhibit a higher rate, up to 40%, of metastasizing to nearby lymph nodes and the ones of tonsils 77–96% [2,3]. In humans, the location of OSCC also plays a significant role in determining the prognosis. SCCs of the tongue tend to metastasize more rapidly than other parts of the oral cavity. This could be attributed to the dense network of lymphatics in the tongue and the movement of tongue muscles, which may facilitate the spread of cancerous cells [4].

In canine populations, oral squamous cell carcinoma (OSCC) is more commonly observed in larger dog breeds, especially those older than seven years. Breeds like English springer Spaniels, Shetland sheepdog, and German shepherds show a higher incidence of OSCC [3]. OSCCs affecting the tongue seem more prevalent in breeds such as Poodles, Labrador Retrievers, and Samoyeds [5] and those affecting the tonsils in German Shepherds [6].

Dogs and cats, unlike laboratory rodents, manifest spontaneous cancers that closely mimic the heterogeneity observed in human tumors. Notably, as household pets, dogs cohabitate in shared environments with humans, displaying clinical manifestations, traits, and biological patterns akin to human cancer. This hints at the potential existence of common risk factors between humans and dogs. However, this relationship remains not fully elucidated due to the limited number of specific studies within Comparative and Evolutionary Oncology (CEO) [7,8]. Pets live integrated lives with their owners, thereby encountering shared environmental and socio-economic elements that could predispose them to cancer. Both pets and humans face similar environmental hazards, including toxins and carcinogens like air pollutants or pesticides in food and water [7,9].

Human OSCC is primarily associated with risk factors such as alcohol consumption, tobacco use, UV radiation exposure, and viral infections like HPV and EBV. Alcohol and tobacco are the more geographically prevalent risk factors [10,11,12]. Additionally, individuals with Fanconi anemia, a rare hereditary disease, display increased susceptibility to OSCC [10,11,12]. OSCC is closely linked to the oral microenvironment, stemming from contact with saliva, an acidic biological fluid derived from salivary gland secretions widely employed in the diagnosis of oral tumors [10,11,12].

Studies in cats have shown that exposure to household tobacco smoke potentially doubles the risk of them developing oral squamous cell carcinoma. Although a direct statistical significance of this correlation has not been conclusively established, there is a noticeable link between tobacco smoke exposure and high expression of p53 protein in feline OSCCs [13]. In dogs, there is no concrete evidence linking oral SCC in dogs with tobacco smoke exposure. Consequently, dogs are being considered as a comparative model for researching OSCCs not linked to alcohol and tobacco, which are about 10–15% of the total OSCC cases in humans [14].

The COX enzyme plays a crucial role in converting arachidonic acid into prostaglandins (PGs) in the body. This enzyme has two forms: COX-1, constitutively expressed in most cells, and COX-2, an inducible variant expressed at high levels in inflamed tissues [15,16,17].

The epidermal growth factor receptor (EGFR), also known as HER1 or erbB1, belongs to the family of tyrosine kinase receptors. It can form heterodimers with other members of the ErbB family, such as ErbB2, ErbB3, and ErbB4 [18,19]. These receptors play crucial roles in fundamental cellular activities like cell proliferation, division, and differentiation. [20].

COX-2 and EGFR are frequently overexpressed in several malignant tumors associated with various diseases [10,15,16,17,21]. When these molecules are overexpressed in tumors, they share functions in several crucial steps, including angiogenesis, apoptosis inhibition, immune response suppression, increased cell proliferation, invasive potential, cell differentiation, and migration [10,15,16,17,21]. Its importance in oncology is remarkable, with reports of its overexpression in various types of human cancer, including colon [22], stomach, breast, lung, esophagus, pancreas, bladder, prostate, and OSCC [22,23,24,25]. In addition, its expression has also been identified in some canine epithelial tumors, such as adenocarcinomas, mammary gland carcinomas, prostate and ovarian tumors, transitional cell carcinoma, and squamous cell carcinoma [19,26]. Recent studies have highlighted an interconnection between the EGFR and COX-2 pathways, with EGFR signaling inducing COX-2 expression and increasing prostaglandin production [27]. Similarly, COX-2-derived prostaglandin E2 (PGE2) can amplify EGFR signaling [26,28,29]. In addition, it has been observed that EGFR inhibition in canine squamous cell carcinomas reduces COX-2 expression, demonstrating the interdependence of these pathways [28,30]. COX-2 and EGFR are promising pharmacological and chemopreventive targets for treating various pathological conditions, including cancer [30].

Thus, they are promising future biomarkers in veterinary oncology due to the importance of both molecules in progression and malignancy, decreased survival and poor tumor prognosis [13,17]. Few studies have been carried out on the expression of these two molecules in canine OSCC [19,28]. Therefore, in this study, we aimed to investigate the importance of these key molecular components, Cox-2 and EGFR, in canine OSCC.

## 2. Materials and Methods

### 2.1. Animals and Tissue Specimens

We included 34 samples of canine tumors, histologically classified as Oral Squamous Cell Carcinoma (OSCC), from the archives of the Histopathology Laboratory of the University of Trás-os-Montes and Alto Douro (UTAD). Portuguese veterinary clinics and hospitals provided these samples. They were excised from 34 dogs and had been previously fixed in 10% formalin and embedded in paraffin.

Clinical data such as age, gender, and breed were recorded for each animal. Obtaining clinical staging or follow-up information for the animals included in the study was not possible.

For microscopic examination, 4 µm-thick tissue sections were stained with hematoxylin and eosin. Each specimen was reviewed by two independent pathologists (IP and JP). Our analysis included all slides and meticulously evaluated all the tumor sections.

The histopathologic diagnosis criteria were based on the internationally recognized classification system for animal tumors established by the World Health [31].

Additionally, ten samples of normal canine oral mucosa were included, and collected in the post-mortem routine examination.

### 2.2. Histopathological Evaluation

Histological grading was determined using a modified version of the multifactorial system developed by Anneroth [32]. The assessed parameters included keratinization/differentiation, nuclear pleomorphism, mitotic count, and the tumor–host relationship, including the invasion pattern, invasion stage, and lymphoplasmacytic infiltration.

The levels of keratinization were stratified based on the proportion of tumor cells exhibiting keratinization, yielding the following grades: I (>50% keratinized cells), II (20–50% keratinized), and III (0–20% keratinized) [citations needed]. Nuclear pleomorphism was classified as I (minimal, >75% mature cells), II (moderate, 50–75% mature cells), or III (marked nuclear pleomorphism, <50% mature cells). Mitotic count was measured across ten high-power fields (HPF) and classified as I (0 to 1 mitosis/HPF), II (2 to 3 mitoses/HPF), or III (≥four mitoses/HPF) [32,33].

The pattern of invasion was classified as I (well-defined with pushing borders), II (infiltration by solid cords, bands, and strands), and III (infiltration by small groups, strands, or individual cells). The stage of invasion was categorized as: I (corresponding to carcinoma in situ or questionable invasion), II (apparent invasion limited to the lamina propria), and III (invasion beyond the lamina propria, involving muscle. Lymphoplasmacytic infiltration was evaluated and categorized as I (marked), II (moderate), or III (mild to absent) [32,33].

The sum of these parameters was then used to classify the tumors into three grades: Grade I (scores 5–10) for well-differentiated tumors, Grade II (scores 11–15) for moderately differentiated tumors, and Grade III (scores > 16) for poorly differentiated tumors [32,33]. The presence or absence of emboli was also recorded.

### 2.3. Immunohistochemistry

For immunohistochemistry, sections 3 µm in thickness were used. The primary antibodies included COX-2 (Clone SP21, Transduction Laboratories^®^, Lexington, Kentucky, USA; dilution 1:40; 24 h at 4 °C) and EGFR (clone 31G7, Invitrogen^®^, Paisley, Scotland, UK; dilution 1:100; 45 min at room temperature). These antibodies have been validated in canine tissues [34,35].

Visualization of the primary antibodies was achieved using the NovolinkTM Polymer Detection System (Leica Biosystems^®^, Newcastle, UK), with 3,3′-diaminobenzidine tetrachloride (DAB) as the chromogen, following manufacturer instructions. Subsequently, tissue sections were counterstained with Gill’s hematoxylin and cover-slipped.

The specificity of the staining was confirmed using negative controls (omitting the primary antibody) and positive controls (kidney samples for COX-2 and normal skin and mammary tumor samples for EGFR).

### 2.4. Quantification of Immunolabeling

For Cox-2, immunolabeling was quantified using a semi-quantitative method adapted from [36], based on the percentage of positive tumor cells (extension) and the intensity of staining. The percentage of the positive cells was given scores ranging from 1 to 3 (1 for ≤10%, 2 for 11–50%, 3 for >51%), while the intensity of staining was also scored from 1 to 3 (weak, moderate, and strong). These scores were combined to produce a final score, calculated as the product of extension and intensity, categorizing the samples as Low (score < 6), and high expression (score ≥ 6).

The immunoreactivity of the EGFR antibody was considered positive when membranous staining above the background level in greater than 1% of tumor cells was detected. The intensity of the staining was evaluated as previously described. High expression was considered in cases where the staining of the membrane was of strong intensity [37].

All samples were independently evaluated by two observers (IP and JP), who were blinded to clinical and pathological characteristics, using a Nikon Eclipse E600 microscope coupled with a Nikon DXM1200 digital camera, provided by Nikon Instruments Inc., Melville, NY, USA. A third reviewer (LD) was consulted in cases of inconsistent findings. A consensus discussion was then held to determine the final score.

### 2.5. Statistical Analysis

Statistical analysis was conducted using SPSS software (Statistical Package for the Social Sciences), version 19.0 (IBM SPSS Statistics for Windows, IBM Corp^®^, Armonk, NY, USA). Categorical variables were analyzed using the chi-square test and Fisher’s exact test. A significance level of *p* < 0.05 was considered statistically significant for all associations.

## 3. Results

### 3.1. Clinical Information

Of the animals with tumors, 47.1% (16 cases) were female, while 52.9% (18 cases) were male, with data missing for four animals. The age range of the animals was 1 to 17 years, with a mean age of 10.600 and a standard deviation of 3.2660. The breeds were as follows: 18 cases (52.9%) were of non-specified breed, 2 cases were Poodles, 2 cases were Labrador Retrievers, and there was 1 case each of the following breeds: Beagle, Boxer, Border Collie, Siberian Husky, Pekingese, Pinscher, and Yorkshire Terrier.

### 3.2. Histopathological Classification of the Tumors

The classification, based on the criteria mentioned earlier, resulted in the following distribution: 9 cases (26.5%) were categorized as well-differentiated tumors (grade I), 9 cases (26.5%) as moderately differentiated tumors (grade II), and 16 cases (47.1%) as poorly differentiated tumors (grade III). Vascular emboli were present in 6 tumors.

### 3.3. COX-2 Immunoreactivity

Normal oral mucosa was negative for Cox-2 in all cases. In oral squamous cell carcinomas, immunoreactivity for COX-2 was diffusely and uniformly present in the cytoplasm of tumor cells, with some variability observed across the histological samples, being more intense in invasive areas. Three cases were negative for COX-2 expression. Among the positive cases, 3 showed focal labeling, 12 had multifocal labeling, and 14 (48.3%) displayed diffuse labeling. Regarding labeling intensity, 4 (13.8%) cases showed weak staining, 6 (20.7%) moderate staining, and 19 (65.5%) strong staining.

In well-differentiated tumors (grade I), most cases were either negative (*n* = 5) or showed weak COX-2 intensity (*n* = 1). In contrast, moderately differentiated squamous cell carcinomas (*n* = 9) predominantly had multifocal (22.2%) or diffuse labeling (55.6%), with strong COX-2 expression observed in 6 cases (66.7%) and moderate reactivity in 2 cases (22.2%). Higher-grade tumors, exhibited multifocal labeling (43.8%) or diffuse labeling (50%), with strong intensity in 10 cases (62.5%). The differences in labeling extent (*p* = 0.003) and intensity (*p* = 0.009) between histological grades were statistically significant. No differences were observed in tumors with vascular invasion.

**Figure 1:**
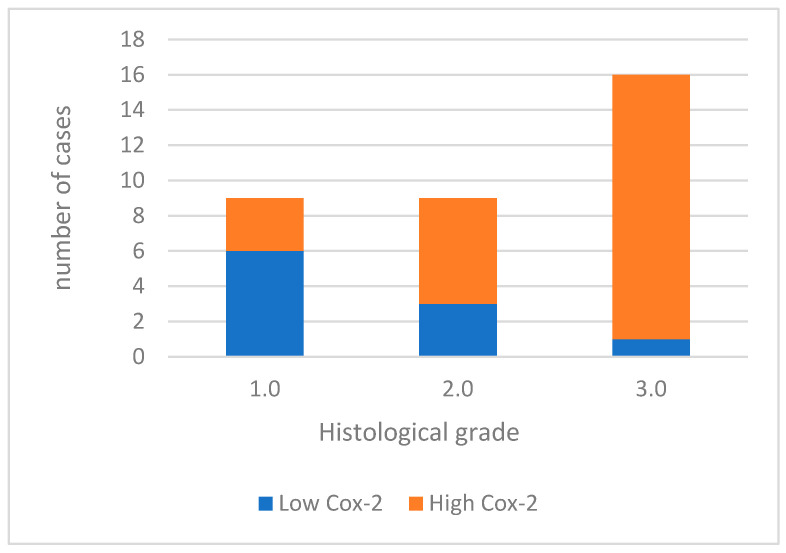
Cox-2 immunoexpression in tumors with different histological grades of malignancy.

**Figure 2:**
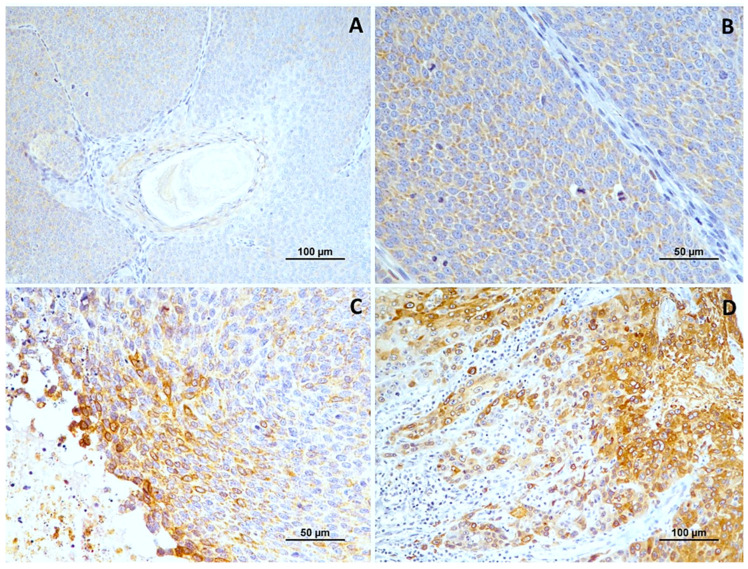
Cox-2 immunoexpression in tumors with different histological grades of malignancy. (**A**) low score in well-differentiated tumors (grade I); (**B**) high score in moderately differentiated tumors (grade II); (**C**,**D**) high score in poorly differentiated tumors (grade III).

For data analysis, COX-2 expression was categorized as low in 10 (29.4%) cases and high in 24 (70.6%) cases (Figure 1). When analyzing the association between COX-2 immunoreactivity and the histological grading of the tumors, a significant correlation was found with higher tumor grading (poorly differentiated tumors) and COX-2 immunoreactivity (*p* = 0.006). Figure 2 shows Cox-2 immunoexpression in tumors with different histological grades of malignancy.

### 3.4. EGFR Immunoreactivity

In normal oral mucosa, EGFR was present in all cases with a moderate membranous reaction. In OSSC, immunoreactivity for EGFR was observed in all cases, with cytoplasmic patterns in 15 tumors (44.1%) and, more frequently, membranous patterns in 19 (55.9%). The intensity of labeling varied, being weak in 8 cases, moderate in 11 cases (32.4%), and strong in 15 cases (44.1%).

Most well-differentiated tumors exhibited a cytoplasmic pattern with weak intensity (71.5%). In contrast, moderately and poorly differentiated squamous cell carcinomas predominantly showed a membranous reaction with strong intensity. The differences in labeling intensity among the histological grade groups were statistically significant (*p* = 0.008). However, no significant differences were observed concerning the location of immunoreactivity.

**Figure 3:**
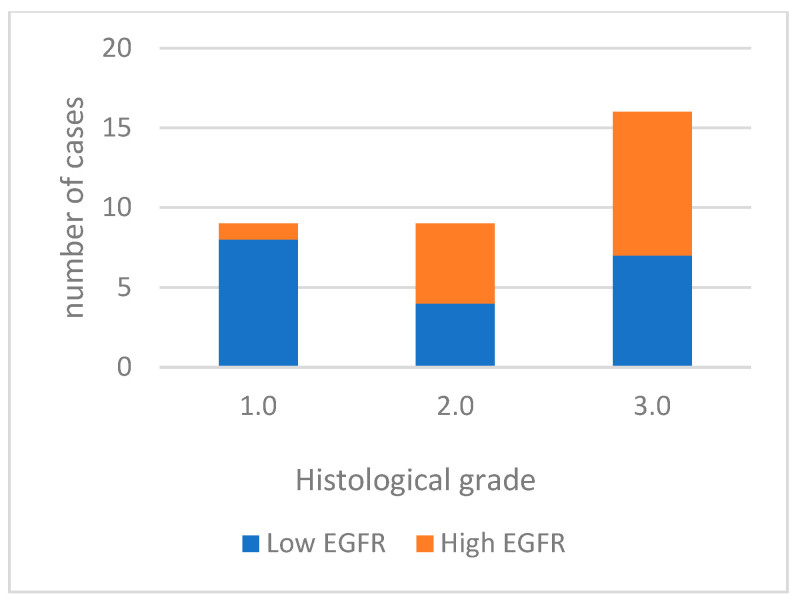
EGFR immunoexpression in tumors with different histological grades of malignancy.

**Figure 4:**
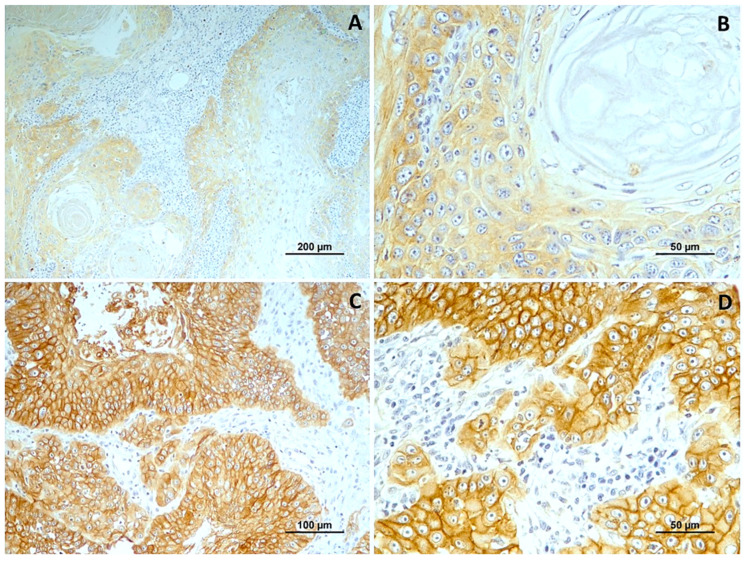
EGFR immunoexpression in tumors with different histological grades of malignancy. (**A**) low score in well-differentiated tumors (grade I); (**B**) low score in a in moderately differentiated tumors (grade II); (**C**,**D**) high score in poorly differentiated tumors (grade III).

For data analysis, EGFR expression was categorized into low expression in 19 cases (55.9%) and high expression in 15 cases (44.1%). Well-differentiated tumors predominantly exhibited low labeling (85.7%). Moderately differentiated tumors had high labeling in 5 out of 9 cases, and most poorly differentiated tumors displayed high labeling (56.3%) (Figure 3). High EGFR expression tends to be more common in tumors with a higher histological grade of malignancy, especially in grade 3.0. However, the differences between tumor grade classes are not statistically significant. (*p* = 0.067). A significant association was noted with vascular invasion (*p* = 0.006). Figure 4 shows EGFR immunoexpression in tumors with different histological grades of malignancy.

### 3.5. Concurrent Cyclooxygenase-2 (COX-2)/Epidermal Growth Factor Receptor (EGFR) Expression

**Figure 5:**
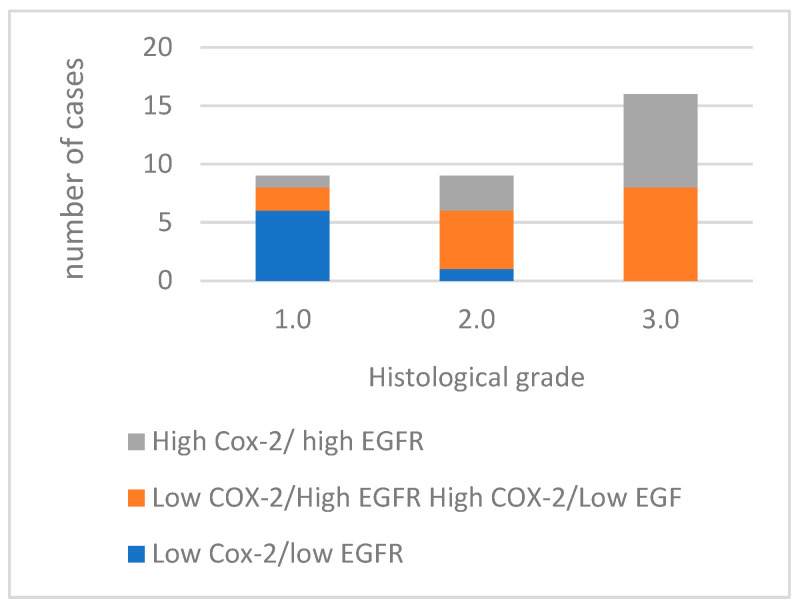
Concurrent expression COX-2 and EGFR in tumors with different histological grades of malignancy.

Of the 34 tumors analyzed, 12 exhibited high immunoreactivity for both COX-2 and EGFR markers. Fifteen cases demonstrated a discordant expression pattern, with either high EGFR and low COX-2 expression, or high COX-2 and low EGFR expression (Figure 5). Furthermore, seven cases showed low immunoreactivity for both COX-2 and EGFR.

Comparing the concurrent expression of COX-2 and EGFR in the histological groups, as the histological grade increases, the number of cases with high expression of both COX-2 and EGFR also increases, with the most noticeable difference occurring in grade 3.0. The absence of cases with low COX-2/low EGFR expression in grades 2.0 and 3.0 is also noted. This association between the concurrent expression of COX-2 and EGFR and the histological grade (*p* = 0.002) and with the presence of vascular invasion (*p* = 0.002) of the tumors is statistically significant.

## 4. Discussion

Oral squamous cell carcinoma (OSCC) stands out as one of the most prevalent malignant tumors of man, making up 1–2% of all malignant tumors worldwide [37,38]. Squamous cell carcinomas (SCCs) are the second most common cancer of the canine oral cavity, resulting in significant morbidity and mortality [39].

In this study, involving 34 cases of -OSCC in dogs, it was observed that the average age of the affected dogs was 10.6 years. No specific gender was more prone to this condition, which is consistent with similar studies [3]. Our study found a higher occurrence of OSCC in mixed breed dogs, although it was also present in various pure breeds, including Poodles, Labrador Retrievers, Beagles, Boxers, Border Collies, Siberian Huskies, Pekingese, Pinschers, and Yorkshire Terriers. Despite the limited sample size, the prevalence of oral cancers in mixed breeds has also been reported in other studies of dogs [14,40]. Contrary to what is generally reported in the literature [3], our study noted that OSCC affected purebred dogs of all sizes, from minor to medium and large. Nevertheless, it is challenging to draw definitive conclusions due to the small sample size and insufficient data on tumor locations and the possibility of mixed breeds being offspring of predisposed breeds.

The main aim of this study was to explore the immunohistochemical expression of COX-2 and EGFR in canine OSCC and assess their correlation with the histological grade of malignancy, as well as to investigate any potential association between these two molecules.

COX-2, an inducible isozyme that plays a crucial role in inflammatory processes, has been associated with malignant diseases [41]. Its overexpression is associated with greater cancer cell growth, increased cell invasion, and an unfavorable prognosis, particularly in canine mammary carcinoma [42]. Our results showed high expression in 70.6% of cases and an association between the histological grade of malignancy and the intensity of Cox-2. These results align with other studies on canine [43,44] and feline [45,46] squamous cell carcinomas. Similar results have also been observed in several other canine tumors, such as mammary tumors [47], melanocytic tumors [48], and rectal and bladder tumors [49], including transitional cell carcinoma [50]. Further research is essential to deepen the relationship between COX-2 expression and indicators of SCC aggressiveness.

Furthermore, in humans, COX-2 has also been implicated in malignancy in several types of cancer, including urothelial tumors [51], laryngeal carcinoma [52], esophageal carcinoma [53] and OSCC [54,55]. Previous studies have highlighted the association COX-2 with angiogenesis and blood vessel formation, and overexpression of COX-2 has been associated with inhibition of apoptosis in tumor cells [56]. The generation of PGE2 by COX-2 has immunosuppressive properties, facilitating the evasion of surveillance mechanisms [57]. The potential use of inhibitors is promising for attenuating resistance to chemotherapy [57].

Thus, our results could suggest that COX-2 inhibitors can treat and increase survival in animals with OSCC. The inhibition of COX-2 activity presents itself as a promising strategy in treating malignant diseases, given the availability of selective and non-selective inhibitors that have exhibited positive effects in high COX-2-expressing cancers. These inhibitors can shift the immune response from supporting tumor growth to destroying it, thereby transforming the tumor microenvironment [58,59]. Piroxicam, a nonsteroidal anti-inflammatory drug (NSAID), has proven beneficial in treating OSCC in dogs. Other potential options, some already licensed for managing pain and inflammation in canines, include mavacoxib [60,61], celecoxib, firocoxib, and enflicoxib [62], among others. However, it is crucial to carefully consider various factors, like the specific subtype of carcinoma, the exact nature and dose of the COX-2 inhibitor, the stage of the tumor, and the effectiveness and practicality of combining these inhibitors with other treatments [63]

EGFR is a cell surface tyrosine kinase fundamental in cell proliferation, angiogenesis, and metastasis—a factor in tumor growth [26,64]. In our study, we observed EGFR overexpression in 56.3% of cases, and we also showed that high EGFR expression tends to be associated with tumors with grade III of malignancy. This aligns with research in human oral [65,66,67] and cutaneous squamous cell carcinomas [68]. The association between EGFR immunoexpression and the degree of malignancy is also consistent with previous studies on several canine cancer [69,70].

In human cancers, EGFR has been associated with a poor prognosis in gastric carcinoma and head and neck SCC [71,72]. Studies in canine cutaneous squamous cell carcinomas have highlighted the role of EGFR in promoting the growth and survival of tumor cells [73]. It is suggested that EGFR may influence prognosis through its direct expression and the modulation of other regulatory molecules that drive tumor growth [74].

In the field of human medicine, anti-EGFR therapies are promising in the treatment of squamous cell carcinoma [71,75,76]. The application of anti-EGFR therapies, including monoclonal antibodies target at the receptor’s surface or tyrosine kinase inhibitors targeting its intracellular domain, has shown encouraging results in canine tumors [77,78,79,80] and oral squamous cell carcinoma in cats, particularly with Cetuximab [77]. However, conclusive evidence regarding their absolute efficacy is still lacking. Our findings underscore the importance of focusing scientific research on developing targeted molecular therapies in veterinary medicine, specifically utilizing tyrosine kinase inhibitors (TKIs) and anti-EGFR monoclonal antibodies. Recent strides in comparative oncology have been promoting the transfer of these small molecule inhibitors and monoclonal antibodies from human to veterinary applications [78]. Despite the potential risk of triggering an immune response against these antibodies, leading to adverse effects and reduced treatment efficacy, research in canine mammary cancer suggests that adaptation is viable. The antibodies retain their affinity for EGFR and their anticancer properties in tumor cell lines, thus addressing this challenge. This assertion is supported by our protein alignment data and the significant similarity between canine and human EGFR genes [77,79,80,81].

In our study, in addition to examining the expression of these two molecules in relation to histopathological characteristics, we investigated the correlation between COX-2 and EGFR in canine OSCC. Our results showed a statistically significant association (*p* = 0.002), and we also showed that the expression of these molecules increased along with the histological grade, in line with previous research in human SCC [82] and canine mammary cancer [35]. Furthermore, we have established a link between these two molecules and vascular invasion, underlining their role in tumor malignancy [83].

A definitive therapy for oral squamous cell carcinoma (OSCC) in dogs remains elusive; directing efforts toward targeting EGFR and COX-2 in veterinary clinical oncology holds promise in unveiling fresh perspectives on cancer biology and the efficacy of advanced targeted therapies.

In summary, the emerging roles of COX-2 and EGFR as promising biomarkers in predicting tumor progression suggest potential avenues for future therapies. Utilizing canine models provides a fresh strategy for advancing cancer treatment. Since dogs encounter cancer at rates similar to humans, share mutual risk factors with their human companions, and exhibit remarkably comparable immune systems, they serve as valuable models for investigating human malignancies and novel biomarkers. This underscores their promising role in cancer research.

The signaling pathways involving COX-2 and EGFR remain inadequately understood in canine OSCC, underscoring the necessity for further research. Future studies should explore additional biomarkers and incorporate methodologies like quantitative PCR (qPCR) to analyze COX-2 and EGFR gene expression and Western blot analysis for protein expression. Additionally, incorporating prognostic studies will provide deeper insights into the clinical relevance of our findings.

## 5. Conclusions

These findings revealed the association of COX-2 and EGFR with malignancy and established a correlation between these two molecules. This study proves pivotal in advancing our understanding of new biomarkers in OSCC. In the future, exploring the expression of these two molecules in relation to dog survival holds promise for further insights. Studying the relationship between risk factors impacting humans and those impacting dogs is crucial. This investigation seeks to uncover potential treatments that could simultaneously address issues in both species. Furthermore, understanding the pathways involved in both can aid in utilizing suitable therapies for these shared conditions.

## Data Availability

The data information can be asked for from the corresponding author.
